# Association of Plasma Vitamin D Metabolites With Incident Type 2 Diabetes: EPIC-InterAct Case-Cohort Study

**DOI:** 10.1210/jc.2018-01522

**Published:** 2018-11-09

**Authors:** Ju-Sheng Zheng, Fumiaki Imamura, Stephen J Sharp, Yvonne T van der Schouw, Ivonne Sluijs, Thomas E Gundersen, Eva Ardanaz, Heiner Boeing, Catalina Bonet, Jesus Humberto Gómez, Courtney Dow, Guy Fagherazzi, Paul W Franks, Mazda Jenab, Tilman Kühn, Rudolf Kaaks, Timothy J Key, Kay-Tee Khaw, Cristina Lasheras, Olatz Mokoroa, Francesca Romana Mancini, Peter M Nilsson, Kim Overvad, Salvatore Panico, Domenico Palli, Olov Rolandsson, Sabina Sieri, Elena Salamanca-Fernández, Carlotta Sacerdote, Annemieke M W Spijkerman, Magdalena Stepien, Anne Tjonneland, Rosario Tumino, Adam S Butterworth, Elio Riboli, John Danesh, Claudia Langenberg, Nita G Forouhi, Nicholas J Wareham

**Affiliations:** 1 Medical Research Council Epidemiology Unit, University of Cambridge, Cambridge, United Kingdom; 2 University Medical Center Utrecht, Utrecht University, Utrecht, Netherlands; 3 Vitas AS, Oslo, Norway; 4 Navarra Public Health Institute, Pamplona, Spain; 5 Navarra Institute for Health Research, Pamplona, Spain; 6 Biomedical Research Center Network of Epidemiology and Public Health, Madrid, Spain; 7 Department of Epidemiology, German Institute of Human Nutrition Potsdam-Rehbruecke, Nuthetal, Germany; 8 Catalan Institute of Oncology–Bellvitge Biomedical Research Institute, Barcelona, Spain; 9 Department of Epidemiology, Murcia Regional Health Council, IMIB-Arrixaca, Murcia, Spain; 10 Center for Research in Epidemiology and Population Health, Faculty of Medicine - University Paris-South, Faculty of Medicine INSERM U1018, University Paris-Saclay, Villejuif, France; 11 Gustave Roussy, Université Paris-Sud, Université Paris-Saclay, Villejuif, France; 12 Department of Clinical Sciences, Lund University, Malmö, Sweden; 13 International Agency for Research on Cancer, Lyon, France; 14 Division of Cancer Epidemiology, German Cancer Research Center, Heidelberg, Germany; 15 University of Oxford, Oxford, United Kingdom; 16 Department of Public Health and Primary Care, University of Cambridge, Cambridge, United Kingdom; 17 Department of Functional Biology, Faculty of Medicine, University of Oviedo, Oviedo, Spain; 18 Public Health Division of Gipuzkoa, BioDonostia Research Institute, San Sebastian, Spain; 19 Department of Public Health, Section for Epidemiology, Aarhus University, Aarhus, Denmark; 20 Dipartimento di Medicina Clinica e Chirurgia, Federico II University, Naples, Italy; 21 Cancer Risk Factors and Life-Style Epidemiology Unit, Institute for Cancer Research, Prevention and Clinical Network, Florence, Italy; 22 Umeå University, Umeå, Sweden; 23 Epidemiology and Prevention Unit, Fondazione IRCSS Istituto Nazionale dei Tumori, Milan, Italy; 24 Escuela Andaluza de Salud Pública, Instituto de Investigación Biosanitaria ibs and Hospitales Universitarios de Granada/Universidad de Granada, Granada, Spain; 25 Unit of Cancer Epidemiology, Città della Salute e della Scienza Hospital-University of Turin and Center for Cancer Prevention, Torino, Italy; 26 National Institute for Public Health and the Environment, Bilthoven, Netherlands; 27 Danish Cancer Society Research Center, Copenhagen, Denmark; 28 Azienda Sanitaria Provinciale di Ragusa, Ragus, Italy; 29 Medical Research Council/ British Heart Foundation Cardiovascular Epidemiology Unit and NIHR Blood and Transplant Research Unit in Donor Health and Genomics, Department of Public Health and Primary Care, University of Cambridge, Cambridge, United Kingdom; 30 School of Public Health, Imperial College London, London, United Kingdom; 31 British Heart Foundation Cambridge Centre of Excellence, Division of Cardiovascular Medicine, Addenbrooke’s Hospital, Cambridge, United Kingdom; 32 Department of Human Genetics, Wellcome Trust Sanger Institute, Wellcome Trust Genome Campus, Hinxton, Cambridge, United Kingdom

## Abstract

**Background:**

Existing evidence for the prospective association of vitamin D status with type 2 diabetes (T2D) is focused almost exclusively on circulating total 25-hydroxyvitamin D [25(OH)D] without distinction between its subtypes: nonepimeric and epimeric 25(OH)D_3_ stereoisomers, and 25(OH)D_2_, the minor component of 25(OH)D. We aimed to investigate the prospective associations of circulating levels of the sum and each of these three metabolites with incident T2D.

**Methods:**

This analysis in the European Prospective Investigation into Cancer and Nutrition (EPIC)–InterAct case-cohort study for T2D included 9671 incident T2D cases and 13,562 subcohort members. Plasma vitamin D metabolites were quantified by liquid chromatography–mass spectrometry. We used a multivariable Prentice-weighted Cox regression to estimate hazard ratios (HRs) of T2D for each metabolite. Analyses were performed separately within country, and estimates were combined across countries using random-effects meta-analysis.

**Results:**

The mean concentrations (SD) of total 25(OH)D, nonepimeric 25(OH)D_3_, epimeric 25(OH)D_3_, and 25(OH)D_2_ were 41.1 (17.2), 40.7 (17.3), 2.13 (1.31), and 8.16 (6.52) nmol/L, respectively. Plasma total 25(OH)D and nonepimeric 25(OH)D_3_ were inversely associated with incident T2D [multivariable-adjusted HR per 1 SD = 0.81 (95% CI, 0.77, 0.86) for both variables], whereas epimeric 25(OH)D_3_ was positively associated [per 1 SD HR = 1.16 (1.09, 1.25)]. There was no statistically significant association with T2D for 25(OH)D_2_ [per 1 SD HR = 0.94 (0.76, 1.18)].

**Conclusions:**

Plasma nonepimeric 25(OH)D_3_ was inversely associated with incident T2D, consistent with it being the major metabolite contributing to total 25(OH)D. The positive association of the epimeric form of 25(OH)D_3_ with incident T2D provides novel information to assess the biological relevance of vitamin D epimerization and vitamin D subtypes in diabetes etiology.

## Teaser

We examined the prospective association of plasma vitamin D metabolites with type 2 diabetes and found that the epimeric form of plasma 25(OH)D_3_ was positively associated with type 2 diabetes risk.

The global burden of type 2 diabetes (T2D) continues to expand, making the prevention of T2D a high public health priority (1). Adequacy of vitamin D status, indicated by circulating 25-hydroxyvitamin D [25(OH)D], has been proposed as a modifiable factor for the prevention of T2D (2, 3). However, previous observational studies reporting the association of 25(OH)D with incidence of T2D exclusively refer to either total 25(OH)D, comprised of 25(OH)D_3_ and 25(OH)D_2_, or to 25(OH)D_3_, but little is known about the associations with T2D for individual 25(OH)D metabolites, including 25(OH)D_2_ and the epimeric form of 25(OH)D_3_ [3-epi-25(OH)D_3_] (4), where an epimer refers to one of a pair of stereoisomers including the nonepimeric and epimeric forms.

Circulating 25(OH)D_2_, which is mainly derived from dietary intake or vitamin supplements, has a low detection rate in general populations (5). Therefore, a large sample size with sufficient incident T2D cases is needed to examine the association of 25(OH)D_2_ with T2D risk, which has not been reported so far. The epimeric form 3-epi-25(OH)D_3_ has been recognized only recently as a product of epimerization of the nonepimeric 25(OH)D_3_ (4, 6, 7), although the enzyme for the epimerization step and also the biochemical roles of 3-epi-25(OH)D_3_ remain unknown. In a prior study in Switzerland (8), 3-epi-25(OH)D_3_ as a proportion of total 25(OH)D_3_ [3-epi-25(OH)D_3_ plus nonepimeric 25(OH)D_3_] was positively associated with incident insulin resistance, although its association with incident T2D has not been reported so far. As well as lack of clarity about their associations with incident T2D, the correlates of 3-epi-25(OH)D_3_ and 25(OH)D_2_ across European countries are largely unknown, with previous investigations being limited to a single study or country, including several US cohorts (5, 9–11).

Therefore, the aims of this study were to: (i) investigate the correlates of plasma concentrations of nonepimeric 25(OH)D_3_, 3-epi-25(OH)D_3_, and 25(OH)D_2_ across eight European countries; and (ii) examine the associations with incident T2D for the three 25(OH)D metabolites, as well as total 25(OH)D for comparison with previous literature. We hypothesized that 3-epi-25(OH)D_3_ would be associated with higher risk of T2D given the prior findings (8) and that nonepimeric 25(OH)D_3_, 25(OH)D_2_, and total 25(OH)D would be associated with a lower risk of T2D.

## Materials and Methods

### Study design

The current analysis used data from the European Prospective Investigation into Cancer and Nutrition (EPIC)–InterAct case-cohort study (12), conducted in eight European countries. Briefly, a total of 12,403 T2D cases were ascertained and verified from among 340,234 participants of the EPIC cohort study with 3.99 million person-years of follow-up (1991 to 2007). A subcohort was created by randomly selecting 16,835 individuals from those with available stored blood and buffy coat, stratified by center. A total of 16,154 participants remained in the subcohort after exclusion of those with prevalent diabetes (n = 548) and unknown diabetes status (n = 129). There were 778 overlapping incident T2D cases in the subcohort, which is a feature of the case-cohort design. For this analysis, we excluded individuals with no plasma samples available (n = 4969) or with hemolyzed samples (n = 32), or samples that were too low in volume for analysis (n = 49) or failed in biochemical analysis (n = 78). Therefore, our final sample for analysis included 22,651 participants with measurement of at least one 25(OH)D metabolite (9671 T2D cases and 13,562 subcohort individuals, with 582 incident T2D cases in the subcohort) (7). All participants provided written informed consent, and the study was approved by the local ethics committees in the participating centers and by the Internal Review Board of the International Agency for Research on Cancer.

Ascertainment of incident T2D cases was conducted through a review of multiple sources of evidence, including self-report, linkage to primary care registers, secondary care registers, medication use (drug registers), hospital admissions, and mortality data (12). Information from any follow-up visit or external evidence with a date later than the baseline visit was used. T2D cases in Denmark and Sweden were identified through local and national diabetes and pharmaceutical registers, and they were considered to be verified. To increase the specificity of the case definition for other centers, we sought further evidence for all self-reported cases with independent sources, including individual medical record reviews in some centers (12). Follow-up was censored at the date of diagnosis (31 December 2007) or the date of death, whichever occurred first.

### Measurement of plasma 25(OH)D metabolites and other blood biomarkers

Plasma 25(OH)D metabolites were measured at Vitas AS (Oslo, Norway; a reference laboratory in Europe with a Vitamin D External Quality Assessment Scheme certificate) (13) using liquid chromatography–tandem mass spectrometry. We measured concentrations of nonepimeric 25(OH)D_3,_ 3-epi-25(OH)D_3_, and 25(OH)D_2_ in citrated plasma samples stored at baseline at –196°C (at −150°C in Denmark). Past evidence indicates stability of 25(OH)D and its metabolites in stored serum or plasma samples for >10 years (14). Briefly, 50 µL of human plasma was diluted with 150 µL of isopropanol with deuterium-labeled nonepimeric 25(OH)D_3_ as an internal standard. After thorough mixing (10 minutes) and centrifugation (20 minutes, 4000 rpm at 10°C), an aliquot of 30 µL was injected from the supernatant into the HPLC system (Agilent 1260/1290 liquid chromatograph; Agilent Technologies, Palo Alto, CA) interfaced by atmospheric pressure chemical ionization to an Agilent Technologies 6420 Triple Quad liquid chromatography–tandem mass-spectrometry system operated in multiple reaction monitoring mode. 25(OH)D metabolites were separated on an Ascentis^®^ Express F5 150-mm × 4.6-mm column with 2.7 µM particles, maintained at 20°C. A one-point calibration curve was made from analysis of a natural plasma calibrator, where the value was set by the use of reference material from Chromsystems. The coefficient of variation derived from the quality control samples assessed with the study samples was 8.25% for nonepimeric 25(OH)D_3_, 20.9% for 3-epi-25(OH)D_3_, and 10.1% for 25(OH)D_2_. The lower limits of quantification (LLQs) for nonepimeric 25(OH)D_3_, 3-epi-25(OH)D_3_, and 25(OH)D_2_ were 5 nmol/L, 1 nmol/L, and 3 nmol/L, respectively.

We calculated total 25(OH)D as the sum of nonepimeric 25(OH)D_3_ and 25(OH)D_2_ for comparability with most prior literature. As a secondary definition of total 25(OH)D, we also calculated the sum of three metabolites: nonepimeric 25(OH)D_3_, 3-epi-25(OH)D_3_, and 25(OH)D_2_. The aim of the latter definition was to enable comparability with previous studies that used measurement methods not enabling separation between 3-epi-25(OH)D_3_ and nonepimeric 25(OH)D_3_, and hence 25(OH)D included both D_3_ metabolites (4, 15).

Serum metabolic biomarkers (except for the Umea center in Sweden, where plasma was used) were measured at Stichting Ingenhousz Laboratory (Etten-Leur, Netherlands), including total cholesterol, high-density lipoprotein cholesterol (HDL-C), triglycerides, uric acid, creatinine, aspartate transaminase, alanine transaminase, and *γ*-glutamyltransferase. Low-density lipoprotein cholesterol (LDL-C) was calculated based on the Friedewald formula. Plasma phospholipid fatty acids were measured at Medical Research Council Human Nutrition Research (Cambridge, UK) (16).

### Measurement of diet and other covariates

Dietary information was collected using country- or center-specific self- or interviewer-administered dietary questionnaires, which were developed within each country (17, 18). Baseline physical activity was assessed with a questionnaire (19). Other data were collected by questionnaire on a variety of lifestyle and health-related factors, such as education level, occupational status, smoking status, and menstrual and reproductive history (18).

### Statistical analysis

Statistical analysis was performed using Stata 14 (StataCorp, College Station, TX). We imputed nonepimeric 25(OH)D_3_ values below the LLQ (n = 23) by assigning a random value between 0 and the LLQ, but we did not impute 3-epi-25(OH)D_3_ or 25(OH)D_2_, as 60.3% and 95.5% of their values, respectively, were below the LLQ. We calculated the ratio of 3-epi-25(OH)D_3_ to nonepimeric 25(OH)D_3_, as the ratio reflects the enzyme activity for the epimerization.

We accounted for the seasonality of blood draw using a linear combination of the sine and cosine of the time variable (day of blood draw) (20). All 25(OH)D variables were winsorized using the values representing the first and 99th percentiles of the distribution in the subcohort. We fit a multivariable linear regression model to data from the subcohort to estimate country-specific cross-sectional associations of demographic, lifestyle, and dietary variables and circulating biomarkers with individual 25(OH)D metabolites, except for 3-epi-25(OH)D_3_ and 25(OH)D_2_, where Tobit regression was used owing to the large number of left-censored values below the LLQ (21). The analysis of 25(OH)D_2_ was not stratified by country owing to the small sample size within each country. All metabolites except for nonepimeric 25(OH)D_3_ had skewed distributions and were log transformed. Estimated associations were combined across countries using random-effects meta-analysis.

We used Prentice-weighted Cox regression to estimate country-specific hazard ratios (HRs) for T2D comparing quintiles and per 1 SD (calculated from the subcohort) of each 25(OH)D variable, and then combined these HRs using random-effects meta-analysis. For 25(OH)D_2_, we estimated the HRs from models fit to the overall dataset owing to the small sample sizes with available data within each country. We also estimated the HR of T2D for 3-epi-25(OH)D_3_ or 25(OH)D_2_ below the LLQ compared with the lowest quintile of the exposure. We fit four statistical models: model 1 included age as the underlying timescale, sex, study center, and seasonality; model 2, as model 1 plus smoking status, physical activity, education, alcohol drinking, total energy intake, Mediterranean diet score as an indicator of diet quality, and circulating lipid biomarkers (HDL-C, LDL-C); model 3, as model 2 plus body mass index (BMI); and model 4, as model 3 plus mutual adjustment for the other 25(OH)D metabolites [nonepimeric 25(OH)D_3_, 3-epi-25(OH)D_3_, or 25(OH)D_2_]. We adjusted for HDL-C and LDL-C, rather than total cholesterol, because circulating 25(OH)D was more specifically associated with HDL-C or LDL-C in previous reports (22–24).

We performed several additional analyses based on the most adjusted model 4 for the 25(OH)D metabolites: model 4a, including dietary factors (dietary intake of fish, egg, red meat, dairy products, cereal, poultry, processed meat, offal, margarine, butter, and mushrooms) and vitamin supplement use as covariates; model 4b, excluding people with HbA1c ≥6.5% (or 48 mmol/mol) at baseline or those confirmed as T2D cases within the first 2 years after baseline to assess the potential influence of reverse causality; model 4c, including baseline HbA1c as a covariate to examine the influence of baseline glycemic status; model 4d, including circulating hepatic (alanine transaminase, aspartate transaminase, and *γ*-glutamyltransferase) and renal function markers (creatinine, uric acid) as covariates to examine the influence of liver and kidney function; model 4e, including plasma phospholipid saturated fatty acids and polyunsaturated fatty acids as covariates to examine the influence of plasma fatty acids given that vitamin D is a lipid-soluble vitamin; model 4f, including baseline prevalence of stroke, heart disease, or cancer or family history of diabetes as covariates; and model 4g, among women, we additionally adjusted for hormone therapy use and menopausal status. We also performed an interaction analysis between season and nonepimeric 25(OH)D_3_ on T2D, as well as an analysis stratified by season (in model 4) to investigate whether the association between nonepimeric 25(OH)D_3_ and incident T2D was consistent across seasons.

We investigated the impact of missing covariate data on the results of both the linear and weighted Cox regression analyses using multiple imputation. Analysis was performed in each of 10 imputed datasets that were created using all the covariates from model 4, with the event variable and Nelson-Aalen estimate of cumulative hazard accounting for the case-cohort design; estimates from each imputation were combined using Rubin’s rules (25).

To explore the shape of the association between 25(OH)D metabolites and incident T2D, we applied a multivariable random-effects meta-analysis to combine the estimated country-specific association of plasma 25(OH)D metabolites with incident T2D (based on model 4 above) using a restricted cubic spline (26, 27); for 25(OH)D_2_, the model was fit to the overall dataset rather than by country. As the wide range of 3-epi-25(OH)D_3_ and 25(OH)D_2_ would potentially distort the shape of the associations, we used log-transformed values for these analyses and plotted the HR of T2D by the log-transformed 3-epi-25(OH)D_3_ or 25(OH)D_2_. The *P* values for nonlinearity were calculated using a Wald test of the relevant parameter from the restricted cubic spline model.

Finally, we investigated potential multiplicative interactions of 25(OH)D metabolites with age, sex, BMI, physical activity, hormone therapy use and menopausal status, modeling age, BMI, and physical activity as continuous variables; others (sex, hormone therapy use and menopausal status) as binary. We estimated associations within the relevant subgroups when a significant interaction (*P* < 0.05) was observed.

## Results

### Population characteristics

The mean (SD) plasma concentrations in the subcohort were as follows: plasma total 25(OH)D as a sum of nonepimeric 25(OH)D_3_ and 25(OH)D_2_, 41.1 (17.2) nmol/L; nonepimeric 25(OH)D_3_, 40.7 (17.3) nmol/L; 3-epi-25(OH)D_3_, 2.13 (1.31) nmol/; and 25(OH)D_2_, 8.16 (6.52) nmol/L. The baseline population characteristics by cases and noncases are presented in an online repository (7), and baseline population characteristics in the subcohort by quintiles of plasma 25(OH)D metabolites are presented in Table 1 and an online repository (7). In the subcohort, nonepimeric 25(OH)D_3_ was positively correlated with 3-epi-25(OH)D_3_ (Spearman *r* = 0.44, *P* < 0.001) and negatively correlated with 25(OH)D_2_ (Spearman *r* = −0.30, *P* < 0.001), whereas there was no significant correlation between 3-epi-25(OH)D_3_ and 25(OH)D_2_ (*P* = 0.109) (7).

**Table 1.** Population Characteristics by Quintiles of Plasma Nonepimeric 25(OH)D_3_ and 3-epi-25(OH)D_3_ in the Subcohort of the EPIC-InterAct Study

	Quintiles of Nonepimeric 25(OH)D_3_					Quintiles of 3-epi-25(OH)D_3_					
	Q1 (n = 2712)	Q2 (n = 2712)	Q3 (n = 2713)	Q4 (n = 2712)	Q5 (n = 2713)	Below the LLQ^*a*^ (n = 8049)	Q1 (n = 1102)	Q2 (n = 1103)	Q3 (n = 1102)	Q4 (n = 1103)	Q5 (n = 1103)
Total 25(OH)D^*b*^	19.6 (5.7)	30.7 (3.4)	39.3 (2.9)	48.8 (3.4)	66.8 (11.6)	34.9 (14.5)	41.4 (14)	45.3 (14.6)	47.7 (14.1)	52.2 (14.7)	63.7 (17.5)
Nonepimeric 25(OH)D_3_, nmol/L	18.9 (4.7)	30.3 (2.7)	39 (2.5)	48.6 (3.2)	66.5 (11.5)	34.4 (14.5)	41.1 (14)	45 (14.6)	47.4 (14.1)	52.0 (14.7)	63.5 (17.5)
3-epi-25(OH)D_3_, nmol/L	1.4 (0.4)	1.6 (0.7)	1.7 (0.6)	2.0 (0.9)	2.8 (1.8)		1.1 (0.1)	1.4 (0.1)	1.8 (0.1)	2.3 (0.2)	4.1 (1.7)
Ratio of 3-epi-25(OH)D_3_ to nonepimeric 25(OH)D_3_	0.07 (0.03)	0.05 (0.02)	0.04 (0.02)	0.04 (0.02)	0.04 (0.02)		0.03 (0.01)	0.04 (0.01)	0.04 (0.01)	0.05 (0.02)	0.07 (0.02)
25(OH)D_2_, nmol/L	10.8 (9)	7.8 (4.7)	6.7 (4)	7.1 (4.4)	6.3 (5.9)	8.7 (7)	8.1 (6.9)	7.7 (4.8)	6.6 (3.6)	6.5 (3.5)	6.7 (7.2)
Age, y	52 (8.8)	51.9 (8.8)	51.8 (8.9)	51.5 (9)	50.7 (9.8)	51.7 (9)	51.5 (9.3)	51.8 (8.9)	51.2 (9.3)	51.4 (9)	51.1 (9.4)
BMI, kg/m^2^	26.7 (4.8)	26.7 (4.3)	26.5 (4.2)	25.8 (4)	25.0 (3.6)	26.3 (4.4)	26.0 (4.3)	25.9 (4.1)	25.8 (4.1)	25.9 (3.9)	25.7 (3.7)
Energy intake, kcal/d	2150 (656)	2121 (629)	2151 (633)	2129 (635)	2081 (634)	2111 (633)	2131 (649)	2131 (645)	2125 (635)	2165 (633)	2194 (659)
Sex, %											
Men	35.8	35.8	39.0	38.6	37.5	34.3	35.8	36.5	40.0	44.3	51.8
Women	64.2	64.2	61.0	61.4	62.5	65.7	64.2	63.5	60.0	55.7	48.2
Alcohol consumption, %											
0 g/d	18.7	17.4	16.8	16.3	12.1	18.1	15.7	15.4	14.9	11.8	10.2
>0 to <6 g/d	32.3	33.3	32.6	33.5	38.5	35.2	37.7	32.6	33.8	31.0	26.7
6 to <12 g/d	12.2	14.2	15.1	14.9	15.8	14.0	14.2	16.6	14.9	14.8	14.9
12 to <24 g/d	13.6	15.8	15.6	16.8	15.5	15.1	14.8	15.0	15.8	17.0	17.5
≥24 g/d	22.7	18.9	19.4	18.1	17.8	17.3	17.1	20.0	19.9	25.2	30.2
Mediterranean diet score, %											
Low (score 0–6)	21.3	20.6	19.8	22.2	28.3	21.9	22.5	23.5	25.5	21.6	23.5
Moderate (score 7–10)	44.1	41.8	43.4	43.4	44.3	43.2	43.6	42.0	42.9	44.7	45.1
High (score 11–18)	32.1	35.1	34.8	32.2	25.3	32.7	31.4	32.3	29.1	32.1	28.8
Physical activity, %											
Inactive	29.0	26.5	23.1	21.4	18.9	26.1	21.4	20.9	22.6	19.9	17.0
Moderately inactive	34.4	34.0	34.4	30.6	28.8	33.0	32.9	32.7	30.2	32.5	29.6
Moderately active	20.2	20.6	21.3	24.3	24.6	21.6	21.9	22.7	24.4	22.7	23.8
Active	15.6	17.8	19.6	22.1	25.3	17.6	22.1	22.1	21.7	24.1	28.4
Smoking status, %											
Never	46.6	47.5	47.7	48.3	46.2	48.3	48.3	47.3	47.5	45.9	39.8
Former	21.5	25.6	26.6	28.4	28.9	24.5	27.1	25.6	26.7	29.6	34.5
Current	31.0	25.7	24.3	22.0	23.3	26.2	23.0	25.6	24.5	23.1	23.8
Educational level, %											
None	10.1	10.5	9.3	7.7	5.3	9.2	7.2	7.0	8.9	7.4	8.2
Primary	34.0	32.7	33.2	30.3	28.5	32.3	29.9	33.2	30.6	31.8	29.3
Technical or professional	20.9	21.0	21.6	22.4	24.5	21.4	23.9	23.0	22.2	23.2	23.2
Secondary	13.9	14.9	15.2	15.5	18.3	15.4	15.7	16.0	15.8	16.3	15.5
Higher education	19.4	19.1	18.5	21.7	21.0	19.8	21.0	18.5	20.4	19.1	22.2
Season of blood draw, %											
Winter (December–February)	36.6	28.8	23.1	19.2	14.1	29.1	22.8	21.2	18.9	15.2	9.2
Spring (March–May)	40.6	34.2	28.8	21.5	14.2	31.3	29.9	27.0	24.4	19.7	13.4
Summer (June–August)	8.6	16.8	20.1	24.7	29.2	14.7	18.7	23.1	24.1	30.0	41.1
Autumn (September–November)	14.1	20.0	27.8	34.2	42.4	24.7	28.5	28.6	32.0	34.8	36.4

All values are expressed as mean (SD) or as percentage.

^a^ The LLQs for nonepimeric 25(OH)D_3_ and 3-epi-25(OH)D_3_ are 5 nmol/L and 1 nmol/L, respectively.

^b^ Total 25(OH)D: sum of nonepimeric 25(OH)D_3_ + 25(OH)D_2_.

### Cross-sectional associations of demographic, lifestyle, and dietary variables and circulating biomarkers with plasma 25(OH)D metabolites

We observed seasonal variation for both nonepimeric 25(OH)D_3_ and 3-epi-25(OH)D_3_ across countries with highest levels between July and September, and lowest levels between January and March (*P* < 0.001 for seasonal variation) (7). No consistent pattern of seasonal variation across countries was seen for 25(OH)D_2_.

**Figure 1.**
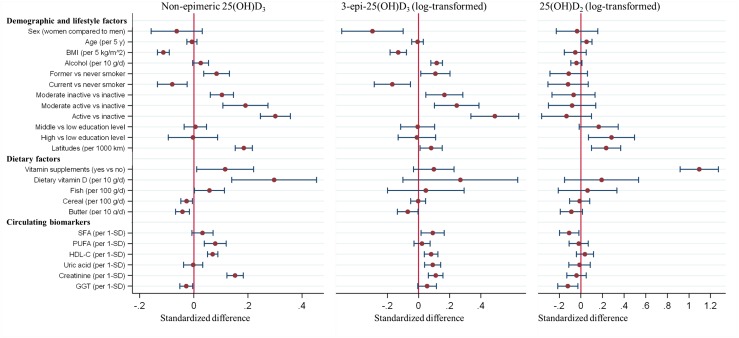
Association of demographic, lifestyle, dietary variables, or circulating biomarkers with plasma 25(OH)D metabolites in the EPIC-InterAct subcohort. The estimates in the figure represent standardized differences in individual 25(OH)D metabolites (in SD unit) per 1 standardized unit/category difference in each demographic, lifestyle, dietary factor, or circulating biomarker. Linear regression or Tobit regression was used to obtain the country-specific estimates of association (except for latitude due to limited study centers in some countries, or 25(OH)D_2_ due to limited sample size), which were combined across countries using random-effects meta-analysis. Only associations that were significant (*P* < 0.05) for at least one metabolite are presented. Estimates for other variables and covariates included in the models are presented in an online repository (7). Results are from complete case analysis, with n = 11,369 for each of the nonepimeric 25(OH)D_3_, 3-epi-25(OH)D_3_, and 25(OH)D_2_. GGT, *γ*-glutamyltransferase; PUFA, polyunsaturated fatty acids; SFA, saturated fatty acids.

Accounting for the influence of seasonal variation and other potential confounders, several demographic, lifestyle, and dietary factors and circulating biomarkers were associated with nonepimeric 25(OH)D_3_ (Fig. 1) (7). Dietary vitamin D intake and vitamin supplements as well as physical activity and serum creatinine were positively associated with nonepimeric 25(OH)D_3_, whereas BMI was inversely associated. Associations with 3-epi-25(OH)D_3_ were generally weaker in magnitude than, but directionally consistent with, nonepimeric 25(OH)D_3_. Alcohol intake and physical activity were both positively associated, whereas BMI was inversely associated with 3-epi-25(OH)D_3_. Vitamin supplements showed the most strongly positive association with 25(OH)D_2_. Alcohol intake was positively associated with the ratio of 3-epi-25(OH)D_3_ to nonepimeric 25(OH)D_3_. Findings from analyses using multiple imputation were similar.

### Association between plasma 25(OH)D metabolites and incident T2D

Table 2 shows that after adjustment for age, sex, center, and seasonality (model 1), there was an inverse association between nonepimeric 25(OH)D_3_ and incident T2D (model 1) that was attenuated after adjustment for diabetes risk factors, circulating lipids, and BMI (models 2 and 3) but became stronger after adjustment for the other vitamin D metabolites. Plasma 3-epi-25(OH)D_3_ was positively associated with T2D in models that adjusted for BMI (model 3) or further adjusted for the other vitamin D metabolites (model 4). Comparing quintiles, a positive association between 3-epi-25(OH)D_3_ and T2D was present for Q5 vs Q1 (Table 2). There was no evidence of an association with T2D comparing 3-epi-25(OH)D_3_ below the LLQ with Q1 in any of the models (Table 2). The association of total plasma 25(OH)D [as the sum of nonepimeric 25(OH)D_3_ and 25(OH)D_2_] with incident T2D was very similar to that of nonepimeric 25(OH)D_3_ across the four models (Table 2). The secondary inclusion of 3-epi-25(OH)D_3_ into the definition of total 25(OH)D produced similar results (7).

**Table 2.** Association of Plasma 25(OH)D Metabolites With Incident T2D: EPIC-InterAct Study

	HR (95% CI)^*a*^						
	Below the LLQ^*b*^	Q1	Q2	Q3	Q4	Q5	Per 1 SD
Total 25(OH)D [nonepimeric 25(OH)D_3_ + 25(OH)D_2_], nmol/L							
Median (range)*^c^*		20.1 (5.68 to <25.9)	30.9 (25.9–35.1)	39.4 (35.1 to <43.7)	48.7 (43.7 to <54.6)	63.6 (54.6 to <118.7)	
No. cases per subcohort		2185/2460	1885/2477	1612/2488	1460/2479	1189/2441	
Model 1		1 (ref)	0.84 (0.71, 0.98)	0.65 (0.57, 0.73)	0.55 (0.48, 0.64)	0.43 (0.39, 0.48)	0.71 (0.68, 0.73)
Model 2		1 (ref)	0.82 (0.70, 0.96)	0.67 (0.59, 0.76)	0.61 (0.53, 0.71)	0.53 (0.46, 0.60)	0.76 (0.73, 0.80)
Model 3		1 (ref)	0.91 (0.75, 1.10)	0.79 (0.67, 0.93)	0.79 (0.64, 0.98)	0.72 (0.60, 0.87)	0.86 (0.81, 0.91)
Model 4		1 (ref)	0.90 (0.74, 1.09)	0.76 (0.65, 0.90)	0.74 (0.61, 0.89)	0.62 (0.54, 0.72)	0.81 (0.77, 0.86)
Nonepimeric 25(OH)D_3_, nmol/L							
Median (range)*^c^*		19.6 (5.30 to <25.5)	30.5 (25.5 to <34.8)	39.0 (34.8 to <43.3)	48.4 (43.3 to <54.4)	63.3 (54.4–118.7)	
No. cases per subcohort		2188/2461	1879/2476	1595/2491	1474/2474	1195/2443	
Model 1		1 (ref)	0.83 (0.72, 0.95)	0.64 (0.57, 0.72)	0.57 (0.50, 0.65)	0.44 (0.39, 0.49)	0.71 (0.69, 0.74)
Model 2		1 (ref)	0.82 (0.71, 0.94)	0.67 (0.58, 0.77)	0.63 (0.55, 0.71)	0.54 (0.47, 0.61)	0.77 (0.74, 0.80)
Model 3		1 (ref)	0.89 (0.76, 1.04)	0.76 (0.63, 0.91)	0.80 (0.66, 0.97)	0.72 (0.61, 0.85)	0.86 (0.82, 0.91)
Model 4		1 (ref)	0.88 (0.75, 1.04)	0.73 (0.61, 0.87)	0.74 (0.62, 0.88)	0.62 (0.54, 0.72)	0.81 (0.77, 0.86)
3-epi-25(OH)D_3_, nmol/L							
Median (range)*^c^*	<1	1.11 (1.00 to <1.25)	1.40 (1.25 to <1.57)	1.76 (1.57 to <1.98)	2.29 (1.98 to <2.74)	3.49 (2.74–15.4)	
No. cases per subcohort	5143/7312	653/1007	678/1000	599/1006	619/1015	639/1005	
Model 1	1.06 (0.95, 1.19)	1 (ref)	1.01 (0.87, 1.17)	0.89 (0.76, 1.03)	0.87 (0.75, 1.01)	0.85 (0.73, 1.00)	0.99 (0.94, 1.04)
Model 2	1.04 (0.92, 1.18)	1 (ref)	1.06 (0.89, 1.26)	0.94 (0.79, 1.11)	0.94 (0.80, 1.11)	0.99 (0.83, 1.17)	1.03 (0.97, 1.09)
Model 3	0.98 (0.83, 1.15)	1 (ref)	1.08 (0.86, 1.35)	0.95 (0.79, 1.14)	1.00 (0.84, 1.20)	1.12 (0.91, 1.37)	1.09 (1.02, 1.17)
Model 4	0.92 (0.79, 1.08)	1 (ref)	1.09 (0.85, 1.38)	0.99 (0.82, 1.19)	1.10 (0.92, 1.31)	1.36 (1.08, 1.71)	1.16 (1.09, 1.25)
25(OH)D_2_, nmol/L							
Median (range)*^c^*	<3	3.36 (3.03 to <3.74)	4.30 (3.74 to <5.06)	6.12 (5.06 to <7.42)	8.74 (7.42 to <11.1)	15.0 (11.1–46.4)	
No. cases per subcohort	7988/11,773	50/111	72/115	70/116	88/114	63/116	
Model 1	1.45 (1.02, 2.05)	1 (ref)	1.16 (0.73, 1.83)	1.07 (0.68, 1.70)	1.32 (0.85, 2.07)	1.01 (0.63, 1.61)	1.00 (0.85, 1.18)
Model 2	1.36 (0.93, 1.98)	1 (ref)	1.04 (0.63, 1.71)	1.12 (0.68, 1.85)	1.44 (0.88, 2.38)	1.02 (0.62, 1.69)	1.02 (0.84, 1.23)
Model 3	1.30 (0.86, 1.96)	1 (ref)	0.90 (0.50, 1.64)	1.18 (0.69, 2.02)	1.18 (0.67, 2.09)	1.08 (0.62, 1.87)	1.03 (0.84, 1.25)
Model 4	1.29 (0.86, 1.95)	1 (ref)	0.93 (0.52, 1.66)	1.15 (0.68, 1.96)	1.10 (0.62, 1.96)	1.00 (0.58, 1.73)	0.94 (0.76, 1.18)

^a^ HRs of T2D comparing quintiles (Q2 to Q5) of 25(OH)D metabolites with Q1 or per 1 SD increase of 25(OH)D metabolites, estimated from country-specific Prentice-weighted Cox regression models; estimates were combined across countries using random-effects meta-analysis. Effect estimates for 25(OH)D_2_ were derived from analysis of the overall EPIC-InterAct data (*i.e.*, not country-specific) owing to limited sample size. 1 SD (calculated from the subcohort) was 17.3 nmol/L for nonepimeric 25(OH)D_3_, 1.31 nmol/L for 3-epi-25(OH)D_3_, and 6.52 nmol/L for 25(OH)D_2_. The present analyses were based on complete case analyses excluding participants with missing covariates based on model 4. The sample size of total cases per subcohort was 8331/12,345 for nonepimeric 25(OH)D_3_, 3188/5033 for 3-epi-25(OH)D_3_ and 343/572 for 25(OH)D_2_. Models were as follows: model 1, adjusted for age (as underlying timescale), sex, center, and seasonality (continuous: sine and cosine function of the day of blood draw); model 2, model 1 plus smoking status (current, former, never), physical activity (inactive, moderately inactive, moderately active, active), education (none, primary, technical or professional, secondary, higher education), alcohol drinking (never, >0 to <6 g/d, 6 to <12 g/d, 12 to <24 g/d, ≥24 g/d), total energy intake (continuous), Mediterranean diet score (low, moderate, high) and plasma lipid biomarkers (continuous: HDL-cholesterol, LDL-cholesterol); model 3, model 2 plus BMI (continuous); model 4, model 3 plus mutual adjustment for the other 25(OH)D metabolites [nonepimeric 25(OH)D_3_ (continuous), 3-epi-25(OH)D_3_ (categorical: below the LLQ, Q1, Q2, Q3, Q4, and Q5), or 25(OH)D_2_ (categorical: below and above the LLQ)].

^a^ The LLQs for 3-epi-25(OH)D_3_ and 25(OH)D_2_ are 1 nmol/L and 3 nmol/L, respectively.

^b^ Median and range of the 25(OH)D metabolites in each quintile in the InterAct subcohort.

There was no evidence of an association between 25(OH)D_2_ and T2D in any of the analyses (Table 2). The ratio of 3-epi-25(OH)D_3_ to nonepimeric 25(OH)D_3_ was positively associated with T2D in all models when comparing the extreme quintile groups (7). There was little evidence of between-country heterogeneity for vitamin D metabolites (7).

Results were largely unchanged in sensitivity analyses that included adjustment for a range of additional factors or that addressed possible reverse causation or applied multiple imputation (7). The association between nonepimeric 25(OH)D_3_ and incident T2D was similar across seasons (no significant interaction was found: *P* interaction = 0.87), with per SD HR 0.85 (0.75 to 0.97), 0.73 (0.62 to 0.86), 0.79 (0.70 to 0.89), and 0.81 (0.73 to 0.89) for winter (December to February), Spring (March to May), Summer (June to August), and Autumn (September to November), respectively.

**Figure 2.**
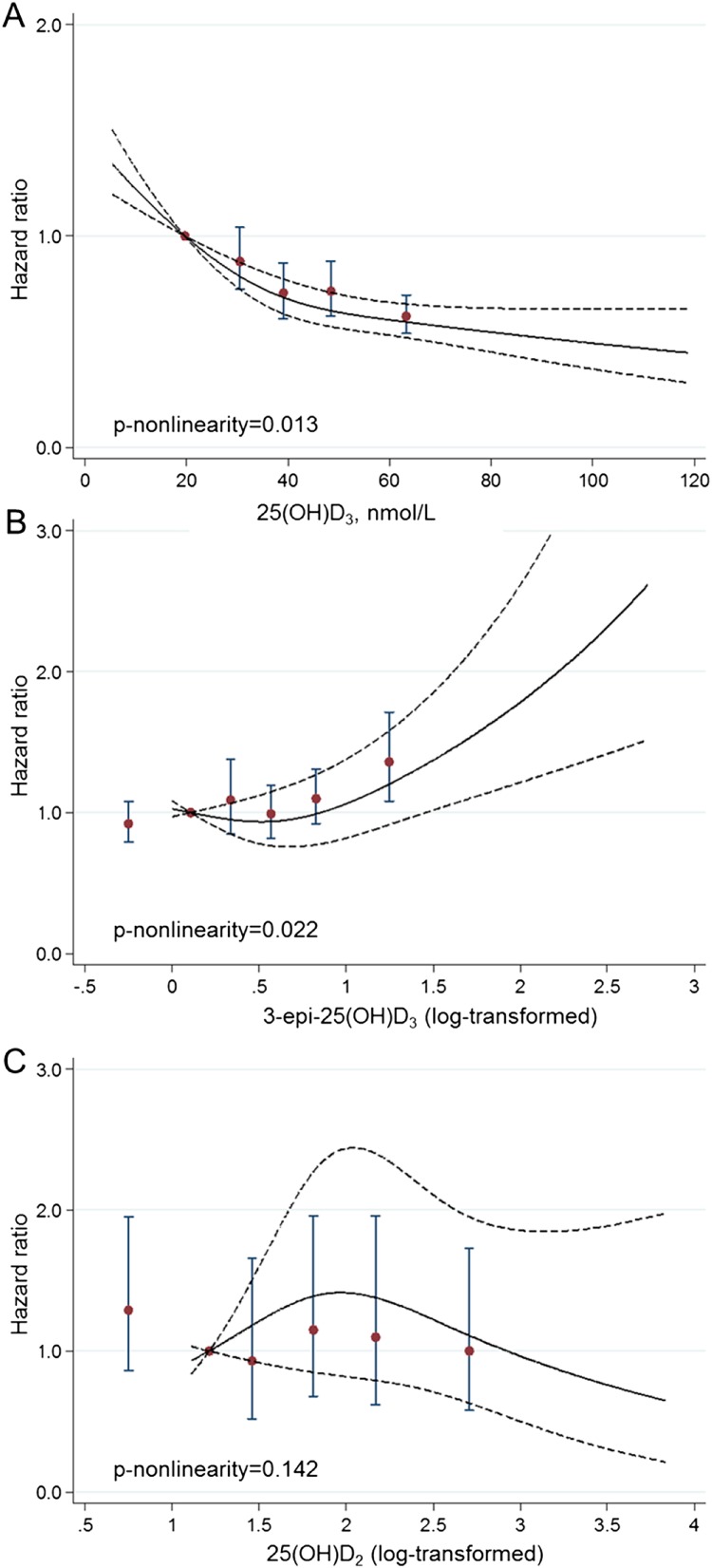
Shape of association between each plasma 25(OH)D metabolite and incident T2D: EPIC-InterAct Study. For 25(OH)D metabolites, restricted cubic spline functions with three knots were used to estimate the association of plasma 25(OH)D metabolites with incident T2D in country-specific [except for 25(OH)D_2_, where all countries were combined] Prentice-weighted Cox regression models, adjusted for potential confounders (*i.e.*, model 4). We then combined the country-specific estimates using a multivariable random-effects meta-analysis. Reference for the HR estimation was the 10th percentile of the corresponding 25(OH)D metabolite: 19.6 nmol/L, 0.10 (equivalent to 1.11 nmol/L), and 1.21 (equivalent to 3.36 nmol/L) for (A) nonepimeric 25(OH)D_3_, (B) 3-epi-25(OH)D_3_ (log transformed), and (C) 25(OH)D_2_ (log transformed), respectively. To enable the dose-response association to be comparable with the quintile results, we also plotted the HR (95% CI) of T2D across the quintiles 2 through 5 of the 25(OH)D metabolites or at the category of below the lower limit of quantification (quintile 1 as reference, location of each plot corresponds to the median level within each quintile).

Cubic spline models suggested a potential nonlinear association with T2D for nonepimeric 25(OH)D_3_ (*P* nonlinearity = 0.013), 3-epi-25(OH)D_3_ (*P* nonlinearity = 0.022), and their ratio (*P* nonlinearity = 0.014) (Fig. 2) (7). A positive association between 3-epi-25(OH)D_3_ and T2D was observed within the fifth quintile when the 3-epi-25(OH)D_3_ was higher than ∼4.48 nmol/L (1.5 in log-transformed value).

There was evidence of interaction (*P* < 0.001) between both nonepimeric 25(OH)D_3_ and 3-epi-25(OH)D_3_ and BMI (7). The inverse association of nonepimeric 25(OH)D_3_ with T2D and the positive association of 3-epi-25(OH)D_3_ with T2D were both stronger among those with a higher BMI.

## Discussion

The multicenter EPIC-InterAct study identified diverse correlates of plasma vitamin D metabolites in epimeric and nonepimeric forms across eight European countries. Specifically, we found that plasma nonepimeric 25(OH)D_3_ was inversely associated with T2D, consistent with findings for total 25(OH)D, whereas 3-epi-25(OH)D_3_ was positively associated with T2D, and 25(OH)D_2_ was not associated with T2D.

Plasma nonepimeric 25(OH)D_3_ was correlated with a variety of lifestyle and dietary factors and circulating biomarkers, confirming previous reports (28, 29). Our finding of higher levels of plasma nonepimeric 25(OH)D_3_ in Northern Europe is consistent with prior reports including European populations (30, 31), but their generalizability was low due to small sample size (<1000 European participants) and limited population representativeness (either in elderly people or in postmenopausal women). Thus, our study with its large sample of European populations provides support for a north-to-south gradient (from high to low) for the distribution of plasma vitamin D_3_ across Europe. The reason for this apparently counterintuitive distribution is probably because of the high intake of dietary vitamin D or vitamin D supplements in Northern European countries (32).

Prior evidence on the correlates of 3-epi-25(OH)D_3_ is limited. In the United States–based NHANES study (n = ∼13,000) (5), serum 3-epi-25(OH)D_3_ was inversely associated with BMI and C-reactive protein, and it was positively associated with vitamin D intake and supplement use. Another United States–based study, ARIC (n = ∼3300) (11), confirmed these associations and additionally found positive associations of serum 3-epi-25(OH)D_3_ with physical activity levels and HDL-C concentrations. We found directionally consistent association of the 3-epi-25(OH)D_3_ with BMI, physical activity, and HDL-C, but no association with dietary vitamin D or supplement use in European populations. Of note, in the current study, alcohol intake was correlated with 3-epi-25(OH)D_3_ or the ratio of 3-epi-25(OH)D_3_ to nonepimeric 25(OH)D_3_, which was not reported in prior studies (5, 11).

For 25(OH)D_2_, owing to the typical low concentration in populations, there have been limited reports of its potential correlates. In a recent Finnish study (33), serum 25(OH)D_2_ was positively associated with a low sunlight exposure period (compared with a high sunlight period) and with use of oral contraceptives. However, the Finnish study did not have detailed dietary or supplement information. Our current findings indicated that vitamin supplements, but not other dietary sources, were the major positive dietary correlates of plasma 25(OH)D_2_.

Several meta-analyses based on observational studies consistently suggested that there was a dose-response inverse association between circulating total 25(OH)D and T2D risk (2, 34, 35), although the sample size of the individual studies included in these meta-analyses was typically small, with studies having between 26 and 829 incident T2D cases (34). Our findings of total 25(OH)D and nonepimeric 25(OH)D_3_, as a major contributor to total 25(OH)D, confirmed results from these previous studies on total 25(OH)D (2, 34, 35) and further confirmed a potential nonlinear association between 25(OH)D and T2D, as suggested previously (36).

No prior study has reported the prospective association between 3-epi-25(OH)D_3_ and T2D risk. Although the epimerization pathway of vitamin D metabolites has been previously observed within *in vitro* studies, the existence of the epimer of vitamin D_3_ in human pediatric and adult populations has only emerged more recently (4), aided by analytic development in resolving the isomeric compounds. To our knowledge, there was only one prior study that assessed 3-epi-25(OH)D_3_ and examined its association with intermediate markers for T2D incidence (8). The study of a Swiss population assessed incident insulin resistance as an outcome and reported no significant association of 3-epi-25(OH)D_3_ but a significant positive association of the proportion of the 3-epi-25(OH)D_3_ in total 25(OH)D_3_, as a sum of epimeric and nonepimeric D_3_ (8). Our present findings for the 3-epi-25(OH)D_3_ and its ratio to nonepimeric 25(OH)D_3_ are novel in relationship to their positive associations with incident T2D. Our findings that further adjustment for 25(OH)D_3_ strengthened the positive association of 3-epi-25(OH)D_3_ supported a potential independent role of the epimer or epimerization process in diabetes etiology.

Although the vitamin D_3_ metabolic pathway has been well characterized (37–39), a physiologic role of 3-epi-25(OH)D_3_ or C-3 epimerization is currently unknown. The secosteroid vitamin D_3_ is derived from the diet and from biosynthesis in the skin (38), undergoing enzymatic hydroxylation to become nonepimeric 25(OH)D_3_ in the liver (39) and then 1,25(OH)_2_D_3_ (the active form of vitamin D_3_) in the kidney (40). While the enzyme, C-3 epimerase, has not been well characterized (41), C-3 epimerization is thought to convert the major vitamin D_3_ metabolites, including 1,25(OH)_2_D_3_ and nonepimeric 25(OH)D_3_, to corresponding epimers. These epimers are thought to have lower biological activity, including lower binding affinity to vitamin D receptor and vitamin D–binding protein, compared with their nonepimeric forms (6), but how this might influence their potential roles in the etiology of T2D is currently unclear. Prior genetic Mendelian randomization studies for 25(OH)D indicated no causal effect of total circulating 25(OH)D (34), but these analyses were unable to dissociate nonepimeric and epimeric 25(OH)D_3_, because a standard assay cannot distinguish between the two (41). The same genetic approach after such decomposition is warranted, because it is possible that the prior null genetic findings reflected both a potentially causal T2D risk–raising effect of epimeric 25(OH)D_3_ (or C-3 epimerase) and risk-lowering effect of the nonepimeric 25(OH)D_3_, thereby leading to overall null findings in the genetic studies.

To our knowledge, no previous study has reported on the association between circulating 25(OH)D_2_ and incident T2D. The lack of research may be due to the technical difficulty in measuring very low concentrations of 25(OH)D_2_ in a large-scale study; hence, to our knowledge, our current study provides the first investigation of 25(OH)D_2_ in diabetes etiology. Although circulating vitamin D_2_ undergoes similar metabolic conversion as vitamin D_3_, the bioactive form of vitamin D_2_ [i.e. 1,25(OH)_2_D_2_] has a much lower binding affinity to vitamin D–binding protein than to vitamin D_3_ and its metabolites (42). The lack of association between 25(OH)D_2_ and incident T2D in our study is therefore consistent with this biological consideration.

The strengths of this study include its large sample size, together with inclusion of measures of multiple 25(OH)D metabolites in epimeric and nonepimeric forms across diverse populations in eight European countries. This enables us to investigate the dietary and lifestyle correlates of these metabolites, as well as their associations with new-onset T2D in a prospective study design. The current study has several limitations. Although 25(OH)D has been shown to be stable in stored samples (14), the stability of the epimeric form has not been confirmed and measurement errors may exist. Nevertheless, observed concentrations of the epimeric 25(OH)D_3_ metabolites were comparable to those of prior studies (4, 11), and potential measurement errors are unlikely to be differential with respect to case status. Our study included predominantly white European-origin populations, therefore justifying the need for further multiethnic investigations, as genetic differences between race/ethnic groups in vitamin D metabolism are clinically important (43). Our observational findings do not imply causality in the observed associations. Future research, including observational studies to confirm our findings as well as experimental studies and Mendelian randomization studies on different 25(OH)D metabolites, is needed to characterize the physiological roles of 25(OH)D metabolites and vitamin D metabolism in the development of T2D.

The current study suggests that nonepimeric 25(OH)D_3_, the major component of total 25(OH)D, is inversely associated, whereas its epimeric form is positively associated with incident T2D. These findings indicate that vitamin D metabolism in relationship to the etiology of T2D is more complex than previously understood, and they provide novel information to assess the biological relevance of vitamin D epimerization and vitamin D subtypes in T2D etiology.

## Abbreviations:

BMI: body mass index
EPIC: European Prospective Investigation into Cancer and Nutrition
HR: hazard ratio
HDL-C: high-density lipoprotein cholesterol
LDL-C: low-density lipoprotein cholesterol
LLQ: lower limit of quantification
T2D: type 2 diabetes
25(OH)D: 25-hydroxyvitamin D
25(OH)D_3_: 25-hydroxyvitamin D_3_
25(OH)D_2_: 25-hydroxyvitamin D_2_
3-epi-25(OH)D_3_: 3-epi-25-hydroxyvitamin D_3_
